# The Successful Treatment of a Case of Prostate Cancer With Brain Metastasis at Diagnosis

**DOI:** 10.7759/cureus.42022

**Published:** 2023-07-17

**Authors:** Ana Vasques, Margarida Lagarto, Marta Pinto, Filipa Ferreira, Ana Martins

**Affiliations:** 1 Medical Oncology, Hospital São Francisco Xavier, Lisbon, PRT; 2 Medical Oncology, Centro Hospitalar Tondela - Viseu, Viseu, PRT; 3 Medical Oncology, Centro Hospitalar de Lisboa Ocidental, Lisbon, PRT

**Keywords:** synchronous, presentation, prognostic, brain metastasis, prostate cancer

## Abstract

Brain metastasis in prostate cancer is quite a rare entity, especially when it manifests at diagnosis. The symptoms are usually non-focal and vary based on the location affected. It is almost always associated with a poor prognosis, with an overall survival of less than a year. The ideal management modality for these patients is not well established but a combination of surgery, radiation, and chemotherapy may be possible options based on the extent and systemic involvement. Brain screening is not done systematically in prostate cancer and more research is needed to understand the outcome this decision would lead to. We report a case of a patient diagnosed with prostate cancer with single metastasis to the brain that manifested as headache and vomiting. The patient was treated with surgery, adjuvant irradiation of the surgical bed, and androgen deprivation therapy. He later underwent intensity-modulated radiation therapy (IMRT) to the prostate and has been remarkably relapse-free for four years.

## Introduction

Prostate cancer is one of the most common malignancies among men worldwide and it usually metastasizes to the bone, lungs, and liver [1-6]. Central nervous system (CNS) involvement is rare, and generally manifests as a single lesion, later in the course of the disease [1-5,7]. Adenocarcinoma is the most frequent histologic subtype based on its incidence rate but the proportion of brain metastasis is higher in other uncommon histologic subtypes such as small cell carcinoma [1-3]. The clinical presentation is predominantly non-focal in most cases, either accompanied by headache, dizziness, behavioral alterations, and memory loss or asymptomatic [1,2,7].

The prognosis is poor in these cases and the median overall survival ranges from one to 12 months, depending on the chosen treatment strategy [1-4,7]. Based on the performance status, systemic disease involvement, and presentation, surgery, radiotherapy, or chemotherapy can be possible treatment options [1-4,7]. Apart from the number, the location of the brain metastasis also appears to have a prognostic impact, with intraparenchymal metastasis having a better prognosis than dural and leptomeningeal variants [3,5,7]. We report a rare case of oligometastatic prostate cancer presenting with headache and vomiting with a surprisingly favorable prognosis after a multimodal management approach.

## Case presentation

**Figure 1:**
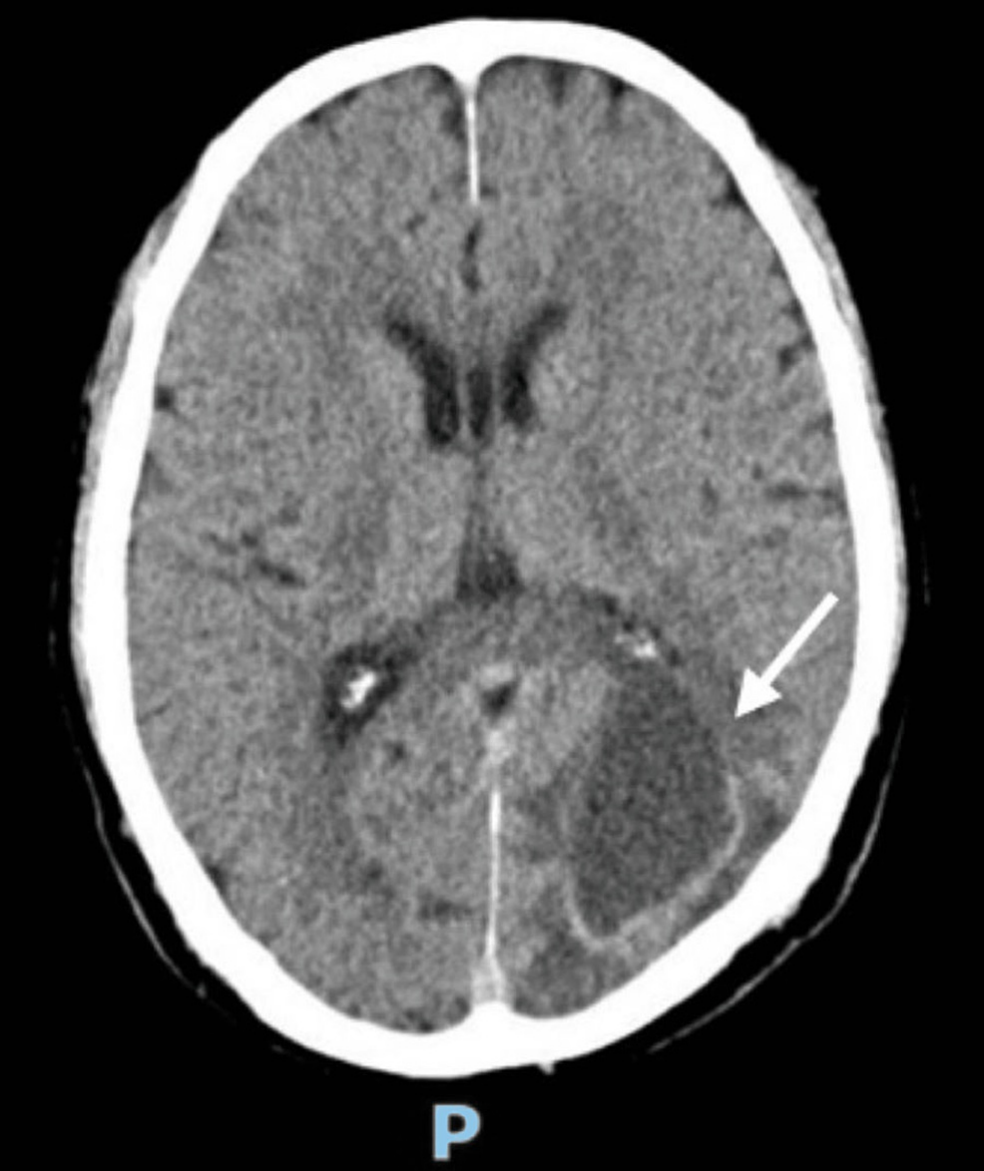
Axial cut of the head CT with visualization of left parieto-occipital lesion before surgery CT: computed tomography

**Figure 2:**
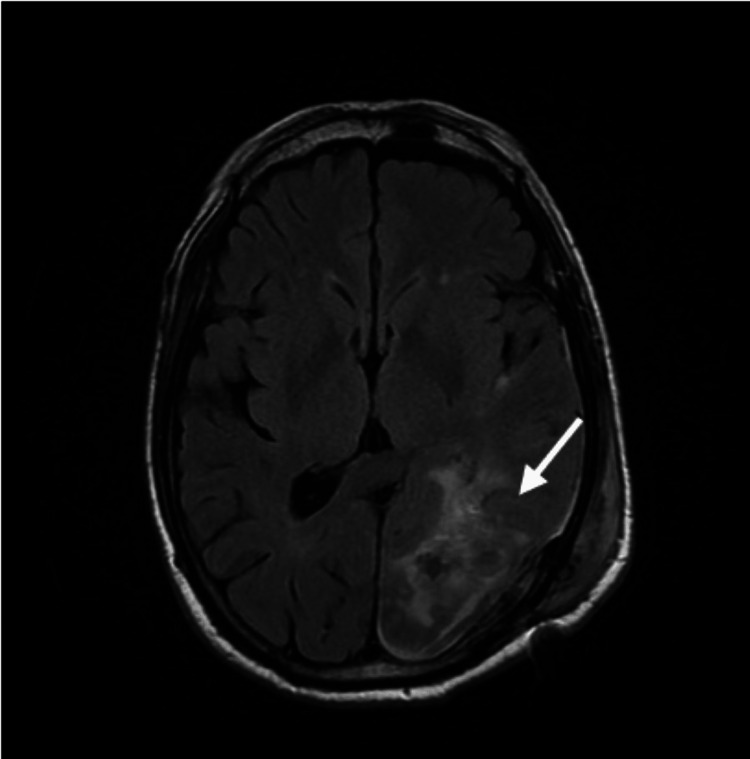
T2 sequence of axial cut of head MRI after surgery MRI: magnetic resonance imaging

A 69-year-old retired male with a history of hypertension and ischemic cardiopathy presented with complaints of headache, nausea, and vomiting in May 2019. The head CT (Figure 1) performed at the emergency department showed a neoplasm in the left parieto-occipital space, with well-defined limits (4.9 x 2.9 cm), accompanied by edema, which was confirmed by MRI. There were no other symptoms, including lower urinary tract symptoms. Body CT and endoscopic studies excluded other sites of malignant disease and the patient subsequently underwent left occipital craniotomy with total macroscopic excision in June 2019. The brain MRI performed one month after the surgery showed total excision of the lesion and serohematic residues in the surgical site (Figure 2). The pathological findings were compatible with brain metastasis of adenocarcinoma of cribriform pattern, positive for prostate-specific antigen (PSA), leading to the diagnosis of brain metastasis from primary prostate carcinoma. After evaluation by the radio-oncology team, the patient underwent treatment with stereotactic radiosurgery (SRS) at a dose of 15 Gy/1 fr, on the surgical bed, in September 2019. There were no significant neurological deficits before or after the surgery and SRS and the patient remained fully independent.

The patient started follow-up with medical oncology and a multiparametric prostate MRI and bone scintigraphy were requested to complete staging. An area with 17 mm at the base of the right hemiprostate slightly spiculated suggested an atypical lesion with invasion of the right seminal vesicle and suspicious adenopathies in the right iliac and obturator chains. There was no evidence of bone metastasis. An endocavitary echo-guided prostate biopsy was performed, in the area of the atypical lesion found on the prostate MRI, but it was negative for malignancy. Androgen deprivation therapy (ADT) with gonadotrophin-releasing hormone agonist (goserelin 10.8 mg every 12 weeks) along with bicalutamide 50 mg id was started in October 2019.

This case was presented at a multidisciplinary reunion, to discuss the benefit of prostate irradiation, despite a negative biopsy for malignancy, and the decision was made to perform prostate radiotherapy. Subsequently, the patient underwent dynamic arc intensity-modulated radiotherapy (VMAT) with simultaneous integrated boost (SIB) at doses of 46 Gy in the iliopelvic ganglia, 50 Gy in the seminal vesicles, and 80 Gy/40 fr in the prostate, from October to December 2020. The PSA decreased from the initial value of 14.2 ng/ml to 0.071 ng/ml (normal value: below 4 ng/ml).

The patient remains under ADT and has shown no evidence of relapse on prostate-specific membrane antigen (PSMA) positron emission tomography (PET) and brain MRI until May 2023.

## Discussion

We presented a unique case of prostate cancer with brain metastasis with a surprisingly good prognosis following management with a multimodal approach. Brain metastasis is not common in prostate cancer, and it very rarely presents synchronously with the primary tumor and with the brain as the only site of metastasis [3]. In this scenario, the neoplasms that typically metastasize to the CNS, such as breast, melanoma, and lung cancer were excluded upfront, and it was only through histology and immunohistochemistry that we were able to discover the primary tumor. The presentation with non-focal brain symptoms, the lack of lower urinary tract symptoms, the tenuous PSA value, and the absence of prostate alterations in the body CT were more consistent with a primary brain tumor and delayed the diagnosis.

The increasing diagnoses of brain metastasis in prostate cancer may be due to more efficient treatments and consequently longer follow-ups, as well as the availability of better diagnosis tools, phenotypic changes of the disease itself, or owing to drug exposure [2,3,5,7]. Radiologic brain screening is not performed systematically in asymptomatic prostate cancer patients although this would allow for an early detection with significant implications for patient prognosis [2-4].

Normally, this patient with oligometastatic prostate cancer with CNS involvement at diagnosis would predictably have a poor prognosis. However, he has remained, to date, without any evidence of relapse in the CNS and with controlled systemic disease, 48 months after diagnosis, challenging all odds. Moreover, the approach of surgery followed by adjuvant irradiation of the surgical bed and systemic ADT turned out to be extremely efficient and led to favorable outcomes in this case.

## Conclusions

This clinical report portrayed a unique case of metastatic prostate cancer, with secondary involvement of the brain exclusively and, remarkably, a great prognosis. Due to the low incidence of brain metastases in prostate cancer and, consequently, their exclusion from clinical trials, there is a dearth of evidence-based data regarding the best approach and treatment. The optimal management option for these patients is not well established. Aggressive treatment, with surgery followed by radiation, when the CNS is the only metastatic site seems ideal, but further research is needed to devise the best treatment strategy for these patients.
